# Synthesis, In Silico, and In Vitro Evaluation of Long Chain Alkyl Amides from 2-Amino-4-Quinolone Derivatives as Biofilm Inhibitors

**DOI:** 10.3390/molecules24020327

**Published:** 2019-01-17

**Authors:** Mariana Paola Espinosa-Valdés, Sara Borbolla-Alvarez, Ana Elena Delgado-Espinosa, Juan Francisco Sánchez-Tejeda, Anabelle Cerón-Nava, Osvaldo Javier Quintana-Romero, Armando Ariza-Castolo, Diego Fernando García-Del Río, Marco A. Loza-Mejía

**Affiliations:** 1 Facultad de Ciencias Químicas, Universidad La Salle-México. Av. Benjamin Franklin 45, Cuauhtémoc, Mexico City 06140, Mexico; mariana-espinosa@lasallistas.org.mx (M.P.E.-V.); sara.borbolla@lasallistas.org.mx (S.B.-A.); ana.delgado@lasallistas.org.mx (A.E.D.-E.); juansanchez@lasallistas.org.mx (J.F.S.-T.); anabelle.ceron@lasalle.mx (A.C.-N.); diegodoku@gmail.com (D.F.G.-D.R.); 2 Departamento de Química Orgánica, Centro de Investigación y de Estudios Avanzados del Instituto Politécnico Nacional (CINVESTAV-IPN), Av. Instituto Politécnico Nacional 2508, Mexico City 07360, Mexico; javo_omnosnoa@hotmail.com (O.J.Q.-R.); aariza@cinvestav.mx (A.A.-C.)

**Keywords:** quorum sensing, biofilm, quinolones, molecular docking, molecular dynamics

## Abstract

Infection from multidrug resistant bacteria has become a growing health concern worldwide, increasing the need for developing new antibacterial agents. Among the strategies that have been studied, biofilm inhibitors have acquired relevance as a potential source of drugs that could act as a complement for current and new antibacterial therapies. Based on the structure of 2-alkyl-3-hydroxy-4-quinolone and *N*-acylhomoserine lactone, molecules that act as mediators of quorum sensing and biofilm formation in *Pseudomonas aeruginosa,* we designed, prepared, and evaluated the biofilm inhibition properties of long chain amide derivatives of 2-amino-4-quinolone in *Staphylococcus aureus* and *P. aeruginosa*. All compounds had higher biofilm inhibition activity in *P. aeruginosa* than in *S. aureus.* Particularly, compounds with an alkyl chain of 12 carbons exhibited the highest inhibition of biofilm formation. Docking scores and molecular dynamics simulations of the complexes of the tested compounds within the active sites of proteins related to quorum sensing had good correlation with the experimental results, suggesting the diminution of biofilm formation induced by these compounds could be related to the inhibition of these proteins.

## 1. Introduction

One of the major health issues is the emergence of multidrug resistant (MDR) bacterial strains. Infection and contamination from MDR bacteria could compromise major surgeries and can be life-threating to children and elderly or immunocompromised persons, increasing the risk of complications and treatment costs. This situation creates a need to intensify the efforts directed at the development of new antibacterial agents with different approaches distinct from current therapies. The study of the intercellular communication systems in bacteria, known as quorum sensing (QS) systems, has emerged as an attractive biotarget for the development of new antibacterials [1,2]. QS is a cell density-dependent chemical signaling process that regulates several changes in gene expression, which lead to phenotypes, including bioluminescence, plasmid transfer, synthesis of secondary metabolites, and biofilm formation [3]. The latter plays an important role in animal and human infections and food contamination, as well as in the implant of medical devices [1,4]. A biofilm is defined as a bacterial community that fixes and grows on surfaces through a polysaccharide matrix [5,6]. It has been reported that between 65–80% of infectious diseases are linked to bacterial communities whose proliferation is related to biofilms and that they are more resistant to host defense mechanisms and antimicrobials by approximately 10–1000-fold compared to nonbiofilm forming cultures [7,8,9]. Thus, the disruption of biofilm formation by quorum-sensing inhibitors (QSIs) represents an attractive option for the development of drugs that act as a complement for current and new therapies, as it has been reported that QSIs increase the susceptibility of bacteria to the action of antimicrobials [1,10,11].

**Figure 1:**
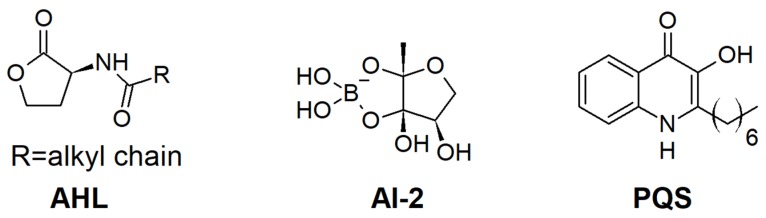
Examples of signaling molecules that mediate quorum sensing.

QS is mediated by several signaling molecules (Figure 1), such as oligopeptides (like AIP-1, generally in Gram-positive bacteria), *N*-acyl homoserine lactones (AHLs, most common in Gram-negative bacteria), furanosyl borate (like AI-2, used by *S. typhimurium*), long chain acid esters, and 2-alkyl-3-hydroxy-4-quinolone in *Pseudonomas aeruginosa*, known as Pseudomonas quinoline signaling (PQS) system [12,13]. The biosynthesis of PQS is mediated by several proteins. In this pathway, anthranilic acid is activated by PqsA, while PqsB, PqsC, and PqsD mediate the conversion to 2-heptyl-4-quinolone (known as HHQ), which is converted into PQS by the participation of PqsH [14]. The transcription of the latter is controlled by LasR, which is one of the major regulators of the virulence factors of *P. aeruginosa*. Thus, inhibition of some of the proteins that participate in this pathway would have an impact on the biofilm formation of *Pseudomonas*. In fact, PqsD and LasR have been evaluated and validated as anti-biofilm targets [15,16,17,18,19,20].

**Figure 2:**
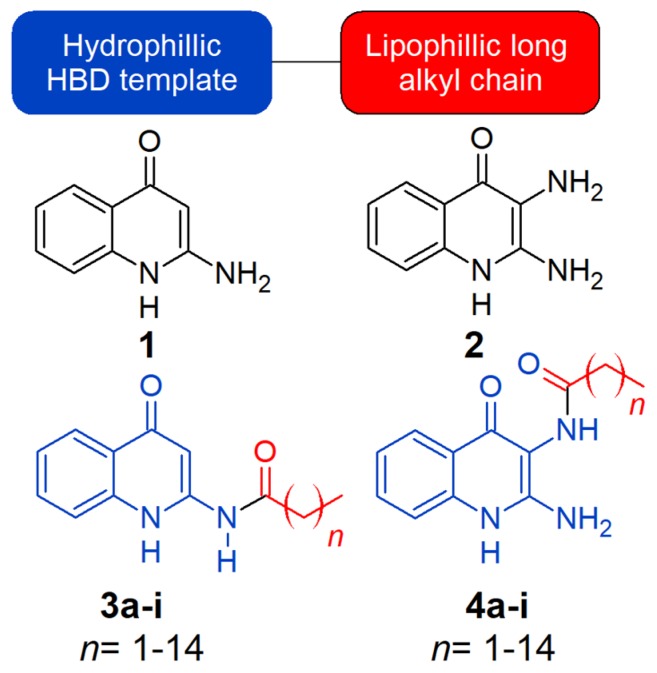
General structural requirements for biofilm inhibitors based on AHL and quinolone derivatives proposed as QSIs.

Some structural requirements have been proposed for the design of biofilm formation inhibitors, which are based primarily on the structure of AHL. It has been proposed that biofilm inhibitors should include a hydrophilic group with hydrogen bond donor (HBD) atoms and a lipophilic alkyl chain (Figure 2). Among the hydrophilic heterocyclic templates that have been studied for the design of biofilm inhibitors [21], some quinolones are described to possess high bioactivity [22,23,24,25,26,27]. We have previously reported a direct route for the synthesis of 2-amino-4-quinolone (compound **1**, Figure 2) and 2,3-diamino-4-quinoline (compound **2**) as key synthons for the preparation of tricyclic compounds [28]. The obvious structural similarity of compounds **1** and **2** with quinolones from the PQS system prompted us to determine if the long chain amide derivatives of **1** and **2** could interfere with bacterial biofilm formation in *P. aeruginosa*. Synthesis, in vitro evaluation of biofilm inhibition, and in silico studies are presented.

## 2. Results

### 2.1. Chemistry

**Scheme 1:**
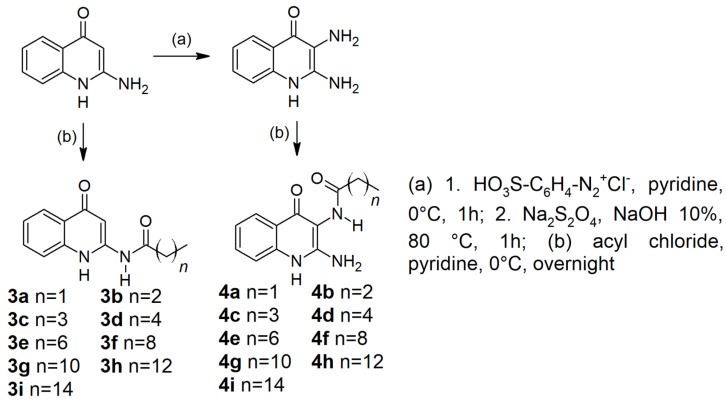
Synthetic route for the preparation of target compounds, **3a-i** and **4a-i**.

Scheme 1 describes the synthetic route for the preparation of the target compounds. The 2-amino-4-quinolone derivatives **1** and **2** were prepared from isatoic anhydride as previously reported [28]. Acylation of **1** and **2** was easily performed from commercially available acid chlorides in pyridine at 0 °C in relatively good yields (70–85%). Variation of these conditions resulted in formation of subproducts (presumably diacylated compounds) or no reaction at all.

**Figure 3:**
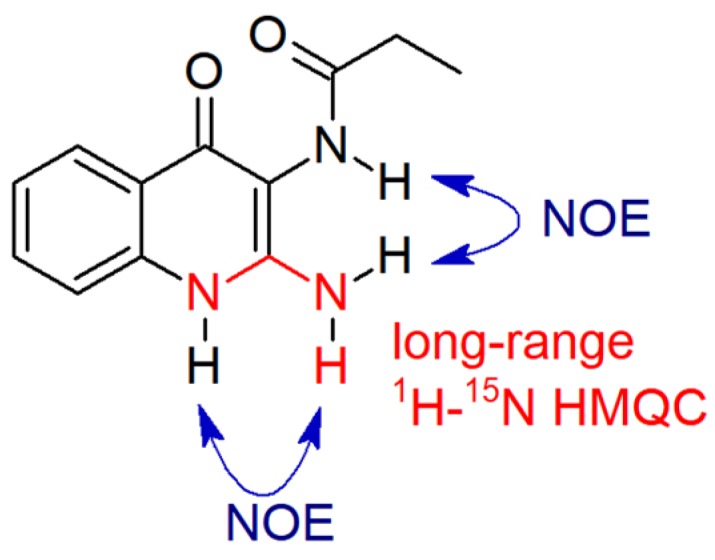
NOE and long-range ^1^H-^15^N-HMQC correlations that confirmed acylation in position 3.

Acylation of the 3-amino group in compound **2** was expected as it seems to be the most reactive group. Selective incorporation of the acyl groups was confirmed by analysis of NMR spectra of compounds **1**, **2** and **4a**. The signal of the protons of 2-amino group in compound **1** appears as a simple signal that integrates for two protons at 6.18 ppm, while in compound **2**, the signal for the protons of the amino group in position 2 appears at 5.99 ppm. For the compounds **4a-i**, a signal that integrates for two protons appears between 5.98 and 6.14 ppm, which allowed us to suppose that the acylation occurred in position 3. Further evidence came from 1D NOE experiments, where the selective irradiation of the protons of the 2-amino group at δ 5.98 ppm in compound **4a** yielded NOEs on the proton of the 3-amide group (δ 11.06 ppm) and the proton bound to the heterocyclic nitrogen atom (δ 8.42 ppm). Additional evidence was provided by the presence of the long-range correlation between the 2-amino group protons and the heterocyclic nitrogen atom in the ^1^H-^15^N long-range HMQC spectra. Figure 3 summarizes these findings. 

### 2.2. Biofilm Inhibition

**Figure 4:**
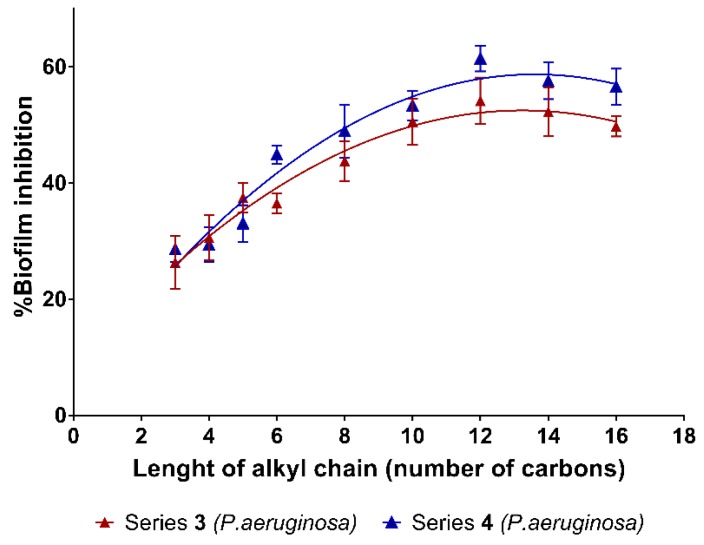
Plot of the percentage of biofilm inhibition in *P.aeruginosa* against the number of carbons in the alkyl chain.

Table 1 shows the results of biofilm inhibition studies of compounds **3a-i** and **4a-i** at 20 M. Results obtained at higher concentrations were considered not reliable due to the poor solubility of the compounds. It is interesting to note that none of the compounds affected bacteria growth, indicating that the decrease of the biofilm formation is not related to bacteriostatic or bactericide activities. An ANOVA analysis using Dunnett’s multiple comparison test showed that compounds with an alkyl chain with 12 carbon atoms or more were the most active and exhibited higher biofilm inhibition activity than tannic acid, which was used as the control [29] (BIC_50_ = 38.4 µM, BIC_50_ = concentration of compound required to reduce biofilm formation to 50%). Compounds **3g** and **4g** also decreased the synthesis of pyocyanin to 68.2% and 62.6% compared to the control, respectively. Values of the percentage efficiency index (PEI), which are derived from the measure of percentage inhibition at a specific concentration divided by the molecular weight [30], are also shown in Table 1. Figure 4 shows the plot of the biofilm inhibition percentage against the alkyl chain length.

### 2.3. Molecular Docking Studies

**Figure 5:**
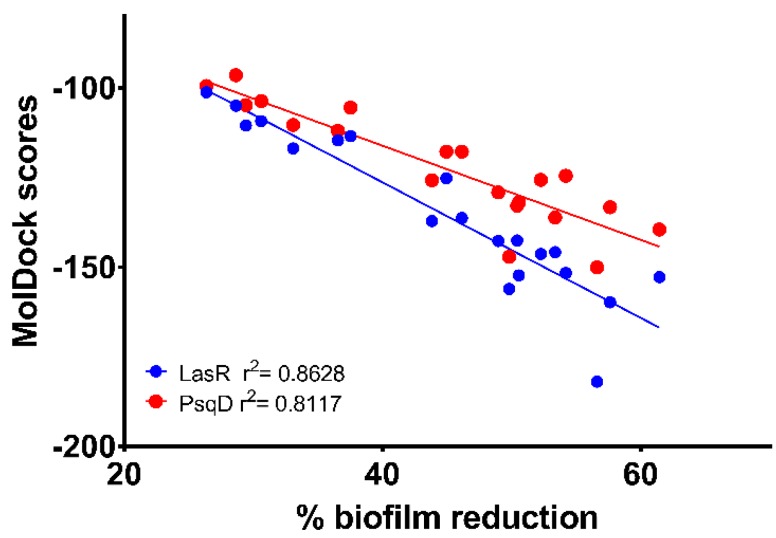
Correlation of the percentage of biofilm inhibition against the docking scores.

Table 2 show the docking scores (MolDock score, lower values are related with higher affinity) obtained during the in silico studies carried out on the LasR and PqsD active sites. Interestingly, MolDock scores had good correlation with biofilm inhibition activity as shown in Figure 5. The values of logP were computed using ChemSketch from ACD/Labs [31] and are also shown in Table 2

### 2.4. Molecular Dynamics Simulations

**Figure 6:**
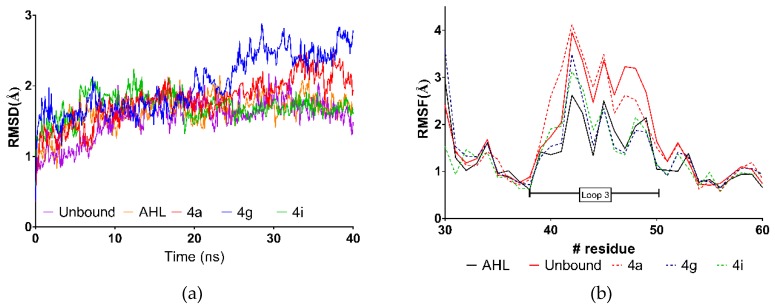
(**a**) RMSD along simulation time plot and (**b**) RMSF of residues of Loop 3 plots generated from data from MD simulations of complexes of **4a**, **4g**, and **4i** with LasR.

**Figure 7:**
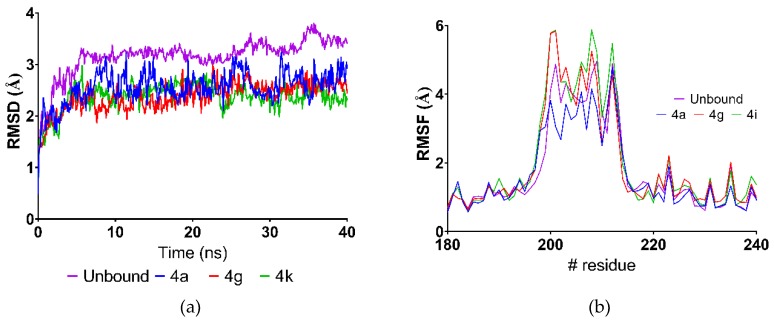
(**a**) RMSD along simulation time plot and (**b**) RMSF of residues along dimer interface (residues 180-240) plots generated from data from MD simulations of complexes of **4a**, **4g**, and **4i** with PqsD.

Molecular dynamics (MD) simulations were carried out to evaluate if the length of the alkyl chain affects the stability of the complexes. Three main parameters were analyzed, the root-median square deviation (RMSD), root-median square fluctuation (RMSF), and the ligand-protein binding energy. The set of Figure 6a,b shows the RMSD and RMSF around Loop 3 of LasR plots of the complexes of **4a**, **4g** and **4i** with LasR, while the set of Figure 7a,b shows the same plots of the complexes of those compounds with PqsD. Table 3 shows the ligand binding energy (LBE) values computed using MM-PBSA approximation. Using these LBE values, an in silico group efficiency (GE) [32,33] was calculated using the following formula:

(1) $$GE_{i}=\frac{\Delta LBE}{\Delta HA}=\frac{LBE_{i}-LBE_{4a}}{HA_{i}-HA_{4a}}$$

where HA is the heavy atom (non-hydrogen) count.

## 3. Discussion

Most of the tested compounds reduced biofilm formation. Table 1 shows the results of biofilm inhibition studies of compounds **3a-i** and **4a-i** at 20 μM. In general, the tested compounds were more active against biofilm formation in *P. aeruginosa* than in *S. aureus.* The most active compounds were those with an alkyl chain of more than 12 carbon atoms (compounds **3g-i** and **4g-i**). This observation coincides with previous reports of biofilm inhibition activities of aminoquinolines, 4-(alkyloxy)-6-methyl-2*H*-pyran-2-one, and aniline derivatives, in which the compounds with an alkyl chain of 12 carbon atoms were the most active [27,34,35], suggesting that alkyl chain length is important for optimal biofilm inhibition. Compounds **3g** and **4g** also decreased the formation of pyocyanin to 68.2% and 62.6% compared to the control, respectively. These findings suggest that these compounds could affect the mechanisms related to virulence of *P. aeruginosa*, including QS.

Derivatives of series **4** were slightly more active than their corresponding analogues of series **3**, but with no significant difference (*p* = 0.44), indicating that the length of alkyl chain is the most important structural factor for biofilm inhibition. One way to evaluate the effect of the increase of the molecular weight (or in our case, the increase in the number of carbons of the alkyl chain) is the measurement of ligand efficiency. We calculated the percentage efficiency index (PEI), which is derived from the measure of percentage inhibition at a specific concentration. Compounds with PEI values higher than 1.5 have a good proportion of potency per molecular weight and are attractive as leads [30]. The PEI values of compounds of series **3** and **4** are shown in Table 2. Compound **4g** exhibited the highest value (1.72) among all the tested compounds, suggesting that it is the most promising molecule in the set. Other parameters of ligand efficiency, like lipophilic ligand efficiency (LLE = pIC_50_ − logP, [36]), could be applied. However, low solubility of most active compounds made the construction of a reliable dose-response curve to calculate the BIC_50_ difficult. This experimental difficulty reveals that low solubility and high logP values (shown in Table 2) are physicochemical factors to consider for the improvement of the bioactivity of this class of compounds. 

The plot of the percentage of biofilm decrease vs the alkyl length suggests a quadratic relationship between these parameters as shown in Figure 4. A parabolic model correlates the number of carbons in the alkyl chain and biofilm inhibition: *y* = −0.2704*x*^2^ + 7.2998*x* + 6.3152, r^2^ = 0.9232, where *x* is the number of carbons in the aliphatic chain and *y* is the biofilm reduction percentage. Parabolic models in QSAR equations are sometimes related to the influence of logP in membrane permeability, which would be a very important aspect to take in consideration due the presence of a double membrane in *P. aeruginosa* and the hydrophobic nature of the tested compounds. However, only a modest correlation was found between biofilm reduction and clogP using parabolic (r^2^ = 0.61) or linear models. We speculated if the positioning of the alkyl chain within the binding site with proteins of QS signaling system could explain the experimental results better. Among the proteins related to the biofilm formation and other virulence factors, we selected LasR and PqsD as potential targets. LasR is one the major regulators of the quorum sensing signaling systems that controls virulence factors and biofilm formation in *P. aeruginosa,* while PqsD is an enzyme needed for biosynthesis of the signaling molecule PQS. 

Figure 5 and Table 2 show the relationship between the docking scores results with biofilm inhibition activity in *P. aeruginosa*. The biofilm inhibition percentage had a good correlation with docking scores calculated during the studies with LasR and PqsD. Analysis of the PDB structure of the complex of AHL within the active site of LasR shows that the hydrophilic lactone moiety forms hydrogen bonds with Trp60 and the amide group forms hydrogen bonds with Tyr56, Asp73, and Ser129, while the 12-carbon chain is accommodated inside a hydrophobic pocket (see Figure S1 in the Supplemental Information). A closer observation of the interaction of the alkyl chain within this pocket shows that residues Gly38, Leu39, and Ala40 form a curve that obliges the aliphatic chain of AHL to make a turn [37]. This turn is significant, as it is the beginning of Loop 3 of LasR and it has been recently reported that the dynamics of Loop 3 (formed by residues between Leu40 and Phe51) are relevant to establish whether a ligand could act as an agonist or antagonist of LasR [38].

**Figure 8:**
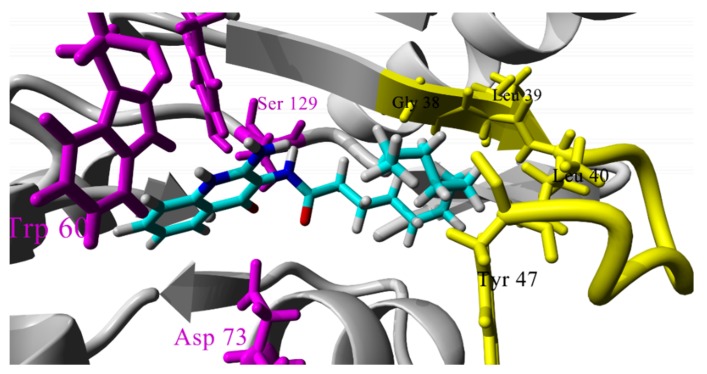
Docking pose for compound **4g** in the active site of LasR. Residues of the hydrophilic pocket that form hydrogen bonds with natural ligand AHL are shown in magenta and those of Loop 3 are in yellow.

Figure 8 shows the docking pose obtained for compound **4g** within the active site of LasR. As expected, the heterocycle occupies almost the same site as the lactone ring of AHL. This occurs for the rest of the compounds of series **3** and **4**. The carbonyl groups of the quinolone and the amide form hydrogen bonds with Tyr64 (Arg61 in case of compounds of series **3**), Thr75, Thr115, and Ser129. The aliphatic chain of **4g** also “makes a turn” close to Leu39 and Ala40 just as AHL. The filling of this hydrophobic pocket in the same mode as AHL is probably important for LasR inhibition as compounds with shorter alkyl chains are less active while compounds with longer chains have torsional strains that make the formation of the complex inside the pocket more difficult.

MD simulations could help to understand and evaluate whether the length of the alkyl chain affects the stability of the complexes. Three main parameters were analyzed, RMSD, RMSF, and the ligand-protein binding energy. Figure 6a shows the RMSD plot of the complexes of **4a**, **4g**, and **4i** with LasR. A smaller value of RMSD indicates higher protein stability. All three complexes exhibited low RMSD variations after 5 ns of simulation, with values lower than 2.8 Å, suggesting that the three complexes are stable. Figure 6b shows the RMSF profile around residues that conform to Loop 3. Around this region, the RMSF values were higher than in the rest of the protein for the five structures analyzed (unbound LasR, LasR-AHL, LasR-**4a**, LasR-**4g**, and LasR-**4i**). Loop 3 exhibited higher flexibility in the absence of any ligands and in the complex with **4a** compared with complexes with AHL, **4g**, and **4i**, particularly around residues 42 to 48, suggesting that the length of the alkyl chain has an important effect on Loop 3. Table 3 shows the ligand binding energy. Interestingly, compound **4a** with the shortest alkyl chain had the better binding, even better than the natural agonist AHL. Compound **4g** and AHL had similar LBE (2.01 KJ/mol and 3.29 KJ/mol), but **4i** had significantly lower LBE, indicating that an increase of the alkyl chain above 12 carbons decreases the efficiency of binding to LasR. It should be noted that with the algorithm implemented in YASARA, the more positive the MM-PBSA binding energy, the better the binding of the ligand.

**Figure 9:**
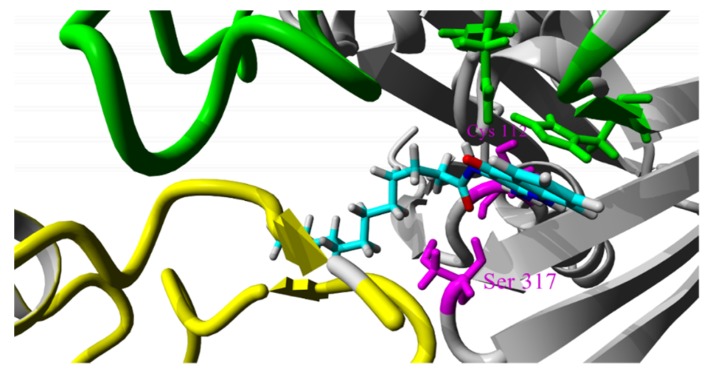
Docking pose of compound **4g** within the active site of PqsD. Key residues for anthranilate binding of Cys112 and Ser317 are shown in magenta.

Analysis of the available structures of PqsD in the complex with some anthranilate derivatives shows that residues Cys112 and Ser317 are important to bind the anthranilate nucleus. Moreover, as part of the proposed mechanism for PqsD, two important “tunnels” have been proposed, one for CoA binding (shown in green in Figure 9) and the other for the β-ketodecanoate (shown in yellow in Figure 9), with kinetics and SPR experiments suggesting a ping-pong mechanism and Acetyl CoA being the first substrate to enter [39,40]. In this mechanism, Ser317 plays a key role, as it is important not only for the binding of the anthranilate ring, but also for binding to the CoA and β-ketodecanoate chains through hydrogen bonding [40]. The docking pose of **4g** within the active site of PqsD shows that the heterocyclic nucleus interacts with Cys112 and Ser317 through the amine group in position 2 and the amide group of position 3 (see Figure S2 in the Supplemental Information), possibly limiting the access of anthranilate to its binding site. All compounds share similar interaction patterns.

To study the effect of the alkyl chain in the stability of the complexes of the tested compounds with PqsD and variations of distance to Ser317 along time, MD simulation of the complexes of **4a**, **4g**, and **4i** were carried out in a similar fashion as those of carried out with LasR. Figure 7a shows the RMSD plot of the complexes of **4a**, **4g**, and **4i** with LasR. From these plots it can be concluded that the three complexes are stable, with minor variations in RMSD. Figure 7b shows the residue RMSF plot. The major variations were observed in the region between residues 195 to 213, which correspond to the hairpin loop of the dimer interface, which coincides with previous studies [40]. Flexibility around this region is increased by the presence of ligands **4g** and **4i**, but decreased by the presence of **4a**, thus it could suggest that in addition to blocking the entrance of anthranilate, this class of compounds could interfere in the formation of the dimer of PqsD. Results of the calculation of the ligand binding energy are shown in Table 3. Compounds **4g** and **4i** clearly exhibit better theoretical binding than **4a**, indicating that inhibition of PqsD is probably a good candidate for the mechanism of action of this class of compounds.

Additionally, group efficiency (GE) was introduced as a metric to evaluate the impact of an added moiety, with higher values indicating a higher potential for development. The values of in silico GE (Table 2), derived from LBE values, suggest that a length of 12 carbon atoms in the alkyl chain has a higher impact than the elongation to 16 atoms. This data suggests that a length of 12 carbon atoms is optimal for biofilm inhibition, and structural modification that leads to compounds with better bioactivity profile should be pursued in the heterocyclic template. 

## 4. Materials and Methods

### 4.1. Chemistry

All reagents were purchased from Sigma-Aldrich and used without further purification. TLC analysis was carried out using silica gel 60 on aluminum foil from Sigma-Aldrich (St. Louis, MO, USA). Melting points were determined on a Fisher-Johns apparatus and are uncorrected. The IR spectra were obtained with a Perkin-Elmer FT IR-670 Plus spectrophotometer in KBr disk. NMR spectra were recorded at room temperature using a Bruker 400 Avance Nanobay (B_0_ = 9.4 T) or a Jeol ECA-500 (B_0_ = 11.7 T), both spectrometers equipped with a 5-mm multinuclear and pulse field gradient probe. The samples were determined in DMSO-*d*_6_ solution. ^1^H-NMR spectra were recorded at 399.7832 MHz and 500.1599 MHz, respectively (spectra width 15 ppm, acquisition time ~2.8 s, pulse width 45°, 16 scans, and recycle delay of 1 s). ^13^C-NMR spectra were recorded at 75.47 MHz or a 125 MHz (spectral with 250 ppm, acquisition time >0.83 s, equivalent 30° pulse duration, scans 3000, and recycle delay of 0.1 s). The following abbreviations are used: s, singlet; br, broad signal; d, doublet; dd, doublet of doublet; t, triplet; m, multiplet. The FAB-MS analyses were obtained on a JEOL Sx102 mass spectrometer. Elemental analyses were performed in a Perkin-Elmer 2400 Series II instrument.

A typical procedure preparation was as follows: 0.12 mmol of the corresponding acid chloride was added to 3 mL of dry pyridine at 0 °C, then 0.1 mmol of the compound **1** or **2** was added, a yellowish suspension was formed, and the mixture was stirred at room temperature overnight. At the end of reaction time, a white precipitate was formed, which was filtered off in vacuum and washed with cold water and cold ethanol to give the target compounds as white solids. 

*N-(4-oxo-1,4-dihydroquinolin-2-yl)propanamide* (**3a**). White solid. mp > 250 °C. IR (cm^−1^): 3208 (br, NH), 2957, 2915, 2850 (CH_2_), 1726, 1655 (C=O). ^1^H-NMR (500.16 MHz) δ 13.35 (s, 1H, NH), 8.15 (br, 1H, NH), 7.94 (dd, *J* = 8.1, 0.7 Hz, 1H, H5), 7.70 (td, *J* = 8.1, 1.4 Hz, 1H, H7), 7.57 (dd, *J* = 8.1, 1.4 Hz, 1H, H8), 7.38 (td, *J* = 8.1, 0.7 Hz, 1H, H6), 6.47 (s, 1H, CH), 2.16 (q, *J* = 7.4 Hz, 2H, CH_2_), 0.93 (t, *J* = 7.4 Hz, 3H, CH_3_). ^13^C-NMR (125.77 MHz) δ 175.76 (NCO), 165.73 (C4O), 156.25 (NCN), 137.76 (C10), 133.32(C7), 124.69 (C6), 123.63 (C5), 117.59 (C8), 116.54 (C9), 92.03 (CH), 27.42 (CH_2_), 9.61 (CH_3_). MS (FAB, *m*/*z*): 217 (M^+^ + 1, 100%); Elemental analysis: C 66.53%, H 5.81%, N 12.59% Calc. for C_12_H_12_N_2_O_2_, C 66.65%, H 5.59%, N 12.96%

*N-(4-oxo-1,4-dihydroquinolin-2-yl)butanamide* (**3b**) White solid. mp > 250 °C. IR (cm^−1^): 3204 (br, NH), 2958, 2910, 2842 (CH_2_), 1725, 1650 (C=O). ^1^H-NMR (500.16 MHz, DMSO-*d*_6_) δ 14.39 (br, 1H, NH) 13.51 (br, 1H, NH), 7.93 (d, *J* = 8.0 Hz, 1H, H5), 7.69 (t, *J* = 8.0 Hz, 1H, H7), 7.57 (d, *J* = 8.0, 1H, H8), 7.37 (t, *J* = 8.0 Hz, 1H, H6), 6.53 (s, 1H, CH), 2.13 (t, *J* = 7.4 Hz, 2H, CH_2_b), 1.45 (sext, *J* = 7.4 Hz, 2H, CH_2_c), 0.82 (t, *J* = 7.4 Hz, 3H, CH_3_d). ^13^C-NMR (125.77 MHz) δ 174.88 (NCO), 165.73 (C4O), 156.32 (NCN), 137.73 (C10), 133.27 (C7), 124.62 (C6), 123.61 (C5), 117.55(C8), 116.57 (C9), 92.06 (C3H), 36.10 (CH_2_ b), 18.44 (CH_2_ c), 14.03 (CH_3_). MS (FAB, *m*/*z*): 231 (M^+^ + 1, 100%); Elemental analysis: C 67.75%, H 6.48%, N 11.93% Calc. for C_13_H_14_N_2_O_2_, C 67.81%, H 6.13%, N 12.17%

*N-(4-oxo-1,4-dihydroquinolin-2-yl)pentanamide* (**3c**) White solid. mp > 250 °C. IR (cm^−1^): 3198 (br, NH), 2967, 2908, 2852 (CH_2_), 1727, 1653 (C=O) ^1^H-NMR (399.78 MHz, DMSO-*d*_6_) δ 13.52 (br, 1H, NH), 8.18 (br, 1H, NH), 7.98 (d, *J* = 8.1 Hz, 1H, H5), 7.73 (dt, *J* = 8.1 Hz, 1H, H7), 7.62 (d, *J* = 8.1, 1H, H8), 7.42 (t, *J* = 8.1 Hz, 1H, H6), 6.53 (s, 1H, CH), 2.18 (t, *J* = 7.4 Hz, 2H, CH_2_b), 1.46 (quint, *J* = 7.4 Hz, 2H, CH_2_c), 1.26 (sext, *J* = 7.4 Hz, 2H, CH_2_d), 0.84 (t, *J* = 7.4 Hz, 3H, CH_3_). ^13^C-NMR (100.52 MHz) δ 174.97 (NCO), 165.68 (C4O), 156.20 (NCN), 137.68 (C10), 133.22 (C7), 124.58 (C6), 123.55 (C5), 117.50(C8), 116.50 (C9), 92.97 (C3H), 33.85 (CH_2_ b), 27.07 (CH_2_), 22.13 (CH_2_), 14.09 (CH_3_). MS (FAB, *m*/*z*): 245 (M^+^ + 1, 100%) Elemental analysis: C 69.05%, H 6.98%, N 11.11% Calc. for C_14_H_16_N_2_O_2_, C 68.83%, H 6.60%, N 11.47%

*N-(4-oxo-1,4-dihydroquinolin-2-yl)hexanamide* (**3d**) White solid. mp > 250 °C. IR (cm^−1^): 3201 (br, NH), 2957, 2918, 2862 (CH_2_), 1724, 1650 (C=O) ^1^H-NMR (399.78 MHz, DMSO-*d*_6_) δ 13.41 (br, 1H, NH), 8.16 (br, 1H, NH), 7.98 (dd, *J* = 8.1, 1.1 Hz, 1H, H5), 7.74 (ddd, *J* = 8.4, 7.9, 1.1 Hz, 1H, H7), 7.62 (dd, *J* = 7.9, 1.1 Hz, 1H, H8), 7.42 (ddd, *J* = 8.4, 8.1, 1.1 Hz, 1H, H6), 6.52 (s, 1H, CH), 2.17 (t, *J* = 7.4 Hz, 2H, CH_2_b), 1.47 (quint, *J* = 7.4, Hz, 2H, CH_2_c), 1.24 (sext, *J* = 7.4 Hz, 2H, CH_2_), 1.22 (sext, *J* = 7.4 Hz, 2H, CH_2_), 0.84 (t, *J* = 7.0 Hz, 3H, CH_3_).^13^C-NMR (100.52 MHz) δ 174.97 (NCO), 165.69 (C4O), 156.19 (NCN), 137.69 (C10), 133.23 (C7), 124.59 (C6), 123.55 (C5), 117.52 (C8), 116.50 (C9), 91.97 (C3H), 34.09 (CH_2_ b), 31.20 (CH_2_), 24.63 (CH_2_), 22.28 (CH_2_), 14.26 (CH_3_).MS (FAB, *m*/*z*): 259 (M^+^ + 1, 100%); Elemental analysis: C 69.65%, H 7.38%, N 10.63% Calc. for C_15_H_18_N_2_O_2_, C 69.74%, H 7.02%, N 10.84%

*N-(4-oxo-1,4-dihydroquinolin-2-yl)octanamide* (**3e**) White solid. mp > 250 °C. IR (cm^−1^): 3203 (br, NH), 2953, 2912, 2857 (CH_2_), 1729, 1655 (C=O). ^1^H-NMR (500.16 MHz, DMSO-*d*_6_) 14.23 (br, 1H, NH), 13.39 (br, 1H, NH), 7.95 (d, *J* = 8.1 Hz, 1H, H5), 7.71 (t, *J* = 8.1 Hz, 1H, H7), 7.58 (d, *J* = 8.1 Hz, 1H, H8), 7.39 (t, *J* = 8.1 Hz, 1H, H6), 6.48 (s, 1H, CH), 2.14 (t, *J* = 7.4 Hz, 2H, CH_2_b), 1.43 (quint, *J* = 6.3 Hz, 2H, CH_2_c), 1.38-1.13 (m, 8H, CH_2_), 0.81 (t, *J* = 7.4 Hz, 3H, CH_3_). ^13^C-NMR (125.77 MHz) δ 175.03 (NCO), 165.76 (C4O), 156.28 (NCN), 137.83 (C10), 133.91 (C7), 124.68 (C6), 123.63 (C5), 117.61 (C8), 116.57 (C9), 92.05 (C3H), 34.20 (CH_2_ b), 29.03 (CH_2_), 28.93 (CH_2_), 25.02 (CH_2_), 24.51 (CH_2_), 22.57 (CH_2_), 14.47 (CH_3_). MS (FAB, *m*/*z*): 287 (M^+^ + 1, 100%); Elemental analysis: C 71.67%, H 7.97%, N 9.59% Calc. for C_17_H_22_N_2_O_2_, C 71.30%, H 7.74%, N 9.78%

*N-(4-oxo-1,4-dihydroquinolin-2-yl)decanamide* (**3f**) White solid. mp > 250 °C. IR (cm^−1^): 3198 (br, NH), 2960, 2920, 2858 (CH_2_), 1721, 1652 (C=O). ^1^H-NMR (500.16 MHz, DMSO-*d*_6_) δ 13.35 (br, 1H, NH), 8.12 (br, 1H, NH), 7.95 (dd, *J* = 8.1, 1.0 Hz, 1H, H5), 7.70 (ddd, *J* = 8.4, 7.1, 1.1 Hz, 1H, H7), 7.58 (d, *J* = 8.1, 1.0 Hz, 1H, H8), 7.38 (ddd, *J* = 8.1, 7.1 1.1 Hz, 1H, H6), 6.46 (s, 1H, CH), 2.14 (t, *J* = 7.4 Hz, 2H, CH_2_b), 1.43 (quint, *J* = 7.0, 6.71 Hz, 2H, CH_2_c), 1.23-1.13 (s, 12H, CH_2_), 0.80 (t, *J* = 7.0 Hz, 3H, CH_3_).^13^C-NMR (125.77 MHz) δ 175.05 (NCO), 165.78 (C4O), 156.25 (NCN), 137.79 (C10), 133.32 (C7), 124.67 (C6), 123.63 (C5), 117.61 (C8), 116.59 (C9), 92.02 (C3H), 34.19 (CH_2_ b), 31.80 (CH_2_), 29.40 (CH_2_), 29.28 (CH_2_), 29.18 (CH_2_), 29.07 (CH_2_), 25.014 (CH_2_), 22.62 (CH_2_), 14.47 (CH_3_). MS (FAB, *m*/*z*): 315 (M^+^ + 1, 100%); Elemental analysis: C 72.59%, H 8.68%, N 8.56% Calc. for C_19_H_26_N_2_O_2_, C 72.58%, H 8.33%, N 8.91%

*N-(4-oxo-1,4-dihydroquinolin-2-yl)dodecanamide* (**3g**). White solid. mp > 250 °C. IR (cm^−1^): 3203 (br, NH), 2957, 2918, 2854 (CH_2_), 1722, 1653 (C=O). ^1^H-NMR (500.16 MHz, DMSO-*d*_6_) 13.23 (br, 1H, NH), 8.08 (br, 1H, NH), 7.94 (d, *J* = 8.1 Hz, 1H, H5), 7.70 (t, *J* = 8.1 Hz, 1H, H7), 7.57 (d, *J* = 8.1 Hz, 1H, H8), 7.38 (t, *J* = 8.1 Hz, 1H, H6), 6.42 (s, 1H, CH), 2.13 (t, *J* = 7.4 Hz, 2H, CH_2_b), 1.43 (m, 2H, CH_2_c), 1.30-1.10 (m, 14H, CH_2_), 0.80 (t, *J* = 6.7 Hz, 3H, CH_3_). ^13^C-NMR (125.77 MHz) δ 175.05 (NCO), 165.77 (C4O), 156.27 (NCN), 137.85 (C10), 133.29 (C7), 124.66 (C6), 123.62 (C5), 117.60 (C8), 116.58 (C9), 92.02 (C3H), 34.20 (CH_2_ b), 31.83 (CH_2_), 29.52 (CH_2_), 29.43 (CH_2_) 29.28 (CH_2_), 29.20 (CH_3_), 29.08 (CH_2_), 28.84 (CH_2_), 25.01 (CH_2_), 22.63 (CH_2_), 14.47 (CH_3_). MS (FAB, *m*/*z*): 343 (M^+^ + 1, 100%); Elemental analysis: C 73.96%, H 9.01%, N 7.87% Calc. for C_21_H_30_N_2_O_2_, C 73.65%, H 8.83%, N 8.18%

*N-(4-oxo-1,4-dihydroquinolin-2-yl)tetradecanamide* (**3h**) White solid. mp: > 250 °C. IR (cm^−1^): 3205 (br, NH), 2956, 2914, 2849 (CH_2_), 1720, 1655 (C=O). ^1^H-NMR (399.78 MHz, DMSO-*d*_6_) δ 13.16 (br, 1H, NH), 8.10 (br, 1H, NH), 7.99 (dd, *J* = 8.1, 1.0 Hz, 1H, H5), 7.75 (ddd, *J* = 8.4, 7.1, 1.4 Hz, 1H, H7), 7.62 (dd, *J* = 8.0, 1.0 Hz, 1H, H8), 7.43 (ddd, *J* = 8.1, 7.1, 1.0 Hz, 1H, H6), 6.44 (s, 1H, CH), 2.18 (t, *J* = 7.4 Hz, 2H, CH_2_b), 1.47 (quint, *J* = 7.4 Hz, 2H, CH_2_c), 1.29-1.17 (m, 20H, CH_2_), 0.84 (t, *J* = 7.0 Hz, 3H, CH_3_).^13^C-NMR (100.52 MHz) δ 175.03 (NCO), 165.75 (C4O), 156.14 (NCN), 137.75 (C10), 133.29 (C7), 124.67 (C6), 123.59 (C5), 117.59 (C8), 116.54 (C9), 91.94 (C3H), 34.12 (CH_2_ b), 31.74 (CH_2_), 29.49 (CH_2_), 29.47 (CH_2_), 29.45 (CH_2_), 29.43 (CH_2_), 29.35 (CH_2_), 29.18 (CH_2_), 29.15 (CH_2_), 28.99 (CH_2_), 24.94 (CH_2_), 22.55 (CH_2_), 14.40 (CH_3_). MS (FAB, *m*/*z*): 371 (M^+^ + 1, 100%); Elemental analysis: C 74.94%, H 9.58%, N 7.21% Calc. for C_23_H_34_N_2_O_2_, C 74.55%, H 9.25%, N 7.56%

*N-(4-oxo-1,4-dihydroquinolin-2-yl)hexadecanamide* (**3i**) White solid. mp > 250 °C. IR (cm^−1^): 3204 (br, NH), 2957, 2918, 2851 (CH_2_), 1726, 1655 (C=O) ^1^H-NMR (399.78 MHz, DMSO-*d*_6_) δ 13.21 (br, 1H, NH), 8.07 (br, 1H, NH), 8.00 (dd, *J* = 8.1, 1.1 Hz, 1H, H5), 7.75 (ddd, *J* = 8.4, 7.1, 1.1 Hz, 1H, H7), 7.61 (d, *J* = 8.4 Hz, 1H, H8), 7.43 (ddd, *J* = 8.1, 7.1, 0.8 Hz, 1H, H6), 6.43 (s, 1H, CH), 2.18 (t, *J* = 7.4 Hz, 2H, CH_2_b), 1.47 (quint, *J* = 7.4 Hz, 2H, CH_2_c), 1.29-1.17 (m, 24H, CH_2_), 0.85 (t, *J* = 7.0 Hz, 3H, CH_3_).^13^C-NMR (100.52 MHz) δ 174.99 (NCO), 165.90 (C4O), 156.15 (NCN), 137.75 (C10), 133.28 (C7), 124.65 (C6), 123.60 (C5), 117.60 (C8), 116.66 (C9), 91.93 (C3H), 34.11 (CH_2_ b), 31.74 (CH_2_), 29.49 (2CH_2_), 29.47 (2CH_2_), 29.47 (CH_2_), 29.43 (CH_2_), 29.36 (CH_2_), 29.19 (CH_2_), 29.16 (CH_2_), 29.00 (CH_2_), 24.95 (CH_2_), 22.56 (CH_2_), 14.41 (CH_3_). MS (FAB, *m*/*z*): 399 (M^+^ + 1, 100%); Elemental analysis: C 75.17%, H 9.97%, N 6.77% Calc. for C_25_H_36_N_2_O_2_, C 75.33%, H 9.61%, N 7.03%

*N-(2-amino-4-oxo-1,4-dihydroquinolin-2-yl)propanamide* (**4a**). White solid. mp > 250 °C. IR (cm^−1^): 3315 (NH_2_), 2960, 2934, 2871 (CH_2_), 1706, 1655 (C=O). ^1^H-NMR (500.16 MHz, DMSO-*d*_6_) δ 11.06 (br, 1H, NH), 8.42 (s, 1H, NH), 7.94 (d, *J* = 9.2 Hz, 1H, H5), 7.47 (t, *J* = 9.2 Hz, 1H, H7), 7.28 (d, *J* = 9.2 Hz, 1H, H8), 7.15 (t, *J* = 9.2 Hz, 1H, H6), 5.98 (s, 2H, NH_2_), 2.30 (t, *J* = 6.9 Hz, 2H, CH_2_), 1.02 (t, *J* = 6.9 Hz, 3H, CH_3_). ^13^C-NMR (100.52 MHz) δ 172.25 (NCO), 171.40 (C4O), 150.28 (NC2N), 148.57 (C3N), 137.66 (C10), 131.66 (C7), 125.62 (C6), 123.35 (C5), 122.21(C8), 116.86 (C9), 28.94 (CH_2_b), 10.19 (CH_3_). MS (FAB, *m*/*z*): 232 (M^+^ + 1, 100%); Elemental analysis: C 62.04%, H 5.89%, N 17.92% Calc. for C_12_H_13_N_3_O_2_, C 62.33%, H 5.67%, N 18.17%

*N-(2-amino-4-oxo-1,4-dihydroquinolin-2-yl)butanamide* (**4b**) White solid. mp > 250 °C. IR(cm^−1^): 3305 (NH_2_), 2956, 2932, 2852 (CH_2_), 1708, 1655 (C=O). ^1^H-NMR (500.16 MHz, DMSO-*d*_6_) δ 10.82 (br, 1H, NH), 8.42 (s, 1H, NH), 7.88 (d, *J* = 8.1 Hz, 1H, H5), 7.74 (t, *J* = 8.0 Hz, 1H, H7), 7.47 (d, *J* = 9.2 Hz, 1H, H8), 7.13 (t, *J* = 9.2 Hz, 1H, H6), 6.12 (s, 2H, NH_2_), 2.13 (t, *J* = 6.9 Hz, 2H, CH_2_), 1.67 (q, *J* = 7.2 Hz, 2H), 0.95 (t, *J* = 7.3 Hz, 3H, CH_3_). ^13^C-NMR (100.52 MHz) δ 172.42 (NCO), 168.75 (C4O), 150.43 (NC2N), 147.57 (C3N), 137.60 (C10), 131.09 (C7), 125.43 (C6), 123.54 (C5), 122.12(C8), 116.80 (C9), 36.22 (CH_2_ b), 18.94 (CH_2_ c), 14.40 (CH_3_). MS (FAB, *m*/*z*): 246 (M^+^ + 1, 100%); Elemental analysis: C 64.01%, H 6.37%, N 16.97% Calc. for C_13_H_15_N_3_O_2_, C 63.66%, H 6.16%, N 17.13%

*N-(2-amino-4-oxo-1,4-dihydroquinolin-2-yl)pentanamide* (**4c**) White solid. mp > 250 °C. IR (cm^−1^): 3305 (NH_2_), 2954, 2921, 2851 (CH_2_), 1705, 1653 (C=O). ^1^H-NMR (500.16 MHz, DMSO-*d*_6_) δ 10.79 (br, 1H, NH), 8.54 (s, 1H, NH), 7.93 (d, *J* = 8.1 Hz, 1H, H5), 7.44 (t, *J* = 8.0 Hz, 1H, H7), 7.28 (d, *J* = 9.2 Hz, 1H, H8), 7.12 (t, *J* = 9.2 Hz, 1H, H6), 6.13 (s, 2H, NH_2_), 2.27 (t, *J* = 7.4 Hz, 2H, CH_2_b), 1.51 (quint, *J* = 7.4 Hz, 2H, CH_2_c), 1.30 (m, *J* = 7.4 Hz, 2H, CH_2_d), 0.86 (t, *J* = 7.4 Hz, 3H, CH_3_). ^13^C-NMR (100.52 MHz) δ 172.72 (NCO), 168.93 (C4O), 150.58 (NC2N), 149.95 (C3N), 137.58 (C10), 130.89 (C7), 125.33 (C6), 123.90 (C5), 122.02(C8), 116.74 (C9), 35.64 (CH_2_ b), 27.95 (CH_2_), 22.48 (CH_2_), 14.43 (CH_3_). MS (FAB, *m*/*z*): 260 (M^+^ + 1, 100%); Elemental analysis: C 65.06%, H 6.85%, N 16.03% Calc. for C_14_H_17_N_3_O_2_, C 64.85%, H 6.61%, N 16.20%

*N-(2-amino-4-oxo-1,4-dihydroquinolin-2-yl)hexanamide* (**4d**) White solid. mp > 250 °C. IR (cm^−1^): 3307 (NH_2_), 2958, 2916, 2870 (CH_2_), 1701, 1654 (C=O). ^1^H-NMR (500.16 MHz, DMSO-*d*_6_) δ 10.71 (br, 1H, NH), 8.51 (s, 1H, NH), 7.66 (d, *J* = 8.1 Hz, 1H, H5), 7.45 (t, *J* = 8.0 Hz, 1H, H7), 7.27 (d, *J* = 9.2 Hz, 1H, H8), 7.13 (t, *J* = 9.2 Hz, 1H, H6), 6.11 (s, 2H, NH_2_), 2.36 (t, *J* = 7.4 Hz, 2H, CH_2_b), 1.56 (m, 2H, CH_2_c), 1.27-1.22 (4H, 2CH_2_), 0.83 (t, *J* = 7.0 Hz, 3H, CH_3_). ^13^C-NMR (100.52 MHz) δ 172.93 (NCO), 164.70 (C4O), 151.61 (NC2N), 147.75 (C3N), 137.92 (C10), 131.85 (C7), 125.48 (C6), 124.54 (C5), 123.80(C8), 116.90 (C9), 35.89 (CH_2_ b), 31.57 (CH2), 25.38 (CH_2_), 22.51 (CH_2_), 14.39 (CH_3_). MS (FAB, *m*/*z*): 274 (M^+^ + 1, 100%); Elemental analysis: C 66.16%, H 7.34%, N 15.13% Calc. for C_15_H_19_N_3_O_2_, C 65.91%, H 7.01%, N 15.37%

*N-(2-amino-4-oxo-1,4-dihydroquinolin-2-yl)octanamide* (**4e**) White solid. mp > 250 °C. IR (cm^−1^): 3308 (NH_2_), 2955, 2934, 2860 (CH_2_), 1702, 1658 (C=O). ^1^H-NMR (500.16 MHz, DMSO-*d*_6_): δ 10.69 (br, 1H, NH), 8.38 (s, 1H, NH), 7.93 (d, *J* = 8.1 Hz, 1H, H5), 7.47 (t, *J* = 8.0 Hz, 1H, H7), 7.20 (d, *J* = 9.2 Hz, 1H, H8), 7.04 (t, *J* = 9.2 Hz, 1H, H6), 6.19 (s, 2H, NH_2_), 2.04 (t, *J* = 7.4 Hz, 2H, CH_2_b), 1.41 (m, 2H, CH_2_c), 1.30-1.04 (m, 8H, CH_2_), 0.79 (t, *J* = 7.4 Hz, 3H, CH_3_). ^13^C-NMR (100.52 MHz) δ 171.53 (NCO), 157.90 (C4O), 150.83 (NC2N), 148.19 (C3N), 137.98 (C10), 130.90 (C7), 126.01 (C6), 124.25 (C5), 122.57 (C8), 116.05 (C9), 34.18 (CH_2_ b), 29.02 (CH_2_), 28.88 (CH_2_), 24.98 (CH_2_), 24.49 (CH_2_), 22.53 (CH_2_), 14.45 (CH_3_). MS (FAB, *m*/*z*): 302 (M^+^ + 1, 100%); Elemental analysis: C 68.01%, H 7.99%, N 13.55% Calc. for C_17_H_23_N_3_O_2_, C 67.75%, H 7.69%, N 13.94%

*N-(2-amino-4-oxo-1,4-dihydroquinolin-2-yl)decanamide* (**4f**) White solid. mp > 250 °C. IR (cm^−1^): 3312 (NH_2_), 2953, 2931, 2862, 2822 (CH_2_), 1702, 1657 (C=O). ^1^H-NMR (500.16 MHz, DMSO-*d*_6_): δ 10.80 (br, 1H, NH), 9.08 (s, 1H, NH), 8.05 (d, *J* = 8.1 Hz, 1H, H5), 7.65 (t, *J* = 8.0 Hz, 1H, H7), 7.28 (d, *J* = 9.2 Hz, 1H, H8), 7.11 (t, *J* = 9.2 Hz, 1H, H6), 6.20 (s, 2H, NH_2_), 2.35 (t, *J* = 7.4 Hz, 2H, CH_2_b), 1.55 (m, 2H, CH_2_c), 1.31-1.14 (m, 12H, CH_2_), 0.82 (t, *J* = 7.0 Hz, 3H, CH_3_). ^13^C-NMR (100.52 MHz) δ 172.45 (NCO), 167.72 (C4O), 150.68 (NC2N), 148.97 (C3N), 137.70 (C10), 129.89 (C7), 124.93 (C6), 123.94 (C5), 122.64 (C8), 116.24 (C9), 35.09 (CH_2_ b), 31.92 (CH_2_), 29.68 (CH_2_), 29.31 (CH_2_), 29.21 (CH_2_), 29.17 (CH_2_), 25.14 (CH_2_), 22.97 (CH_2_), 14.45 (CH_3_). MS (FAB, *m*/*z*): 330 (M^+^ + 1, 100%); Elemental analysis: C 69.61%, H 8.37%, N 12.38% Calc. for C_19_H_27_N_3_O_2_, C 69.27%, H 8.26%, N 12.76%

*N-(2-amino-4-oxo-1,4-dihydroquinolin-2-yl)dodecanamide* (**4g**). White solid. mp > 250 °C. IR (cm^−1^): 3311 (NH_2_), 2951, 2929, 2858 (CH_2_), 1703, 1655 (C=O). ^1^H-NMR (500.16 MHz, DMSO-*d*_6_): δ 10.88 (br, 1H, NH), 8.05 (s, 1H, NH), 7.82 (d, *J* = 8.1 Hz, 1H, H5), 7.65 (t, *J* = 8.0 Hz, 1H, H7), 7.40 (d, *J* = 9.2 Hz, 1H, H8), 7.28 (t, *J* = 9.2 Hz, 1H, H6), 6.19 (s, 2H, NH_2_), 2.05 (t, *J* = 7.4 Hz, 2H, CH_2_b), 1.54 (m, 2H, CH_2_c), 1.31-1.13 (m, 16H, CH_2_), 0.81 (t, *J* = 7.4 Hz, 3H, CH_3_). ^13^C-NMR (100.52 MHz) δ 172.45 (NCO), 167.97 (C4O), 150.68 (NC2N), 148.97 (C3N), 137.70 (C10), 129.35 (C7), 124.97 (C6), 123.94 (C5), 122.64 (C8), 116.4 (C9), 35.10 (CH_2_ b), 32.03 (CH_2_), 29.75 (CH_2_), 29.51 (CH_2_) 29.37 (CH_2_), 29.27 (CH_3_), 29.19 (CH_2_), 28.94 (CH_2_), 25.31 (CH_2_), 22.93 (CH_2_), 14.43 (CH_3_). MS (FAB, *m*/*z*): 358 (M^+^ + 1, 100%); Elemental analysis: C 70.88%, H 9.01%, N 11.53% Calc. for C_21_H_31_N_3_O_2_, C 70.55%, H 8.74%, N 11.75%

*N-(2-amino-4-oxo-1,4-dihydroquinolin-2-yl)tetradecanamide* (**4h**) White solid. mp > 250 °C. IR (cm^−1^): 3314 (NH_2_), 2949, 2930, 2858 (CH_2_), 1702, 1654 (C=O). ^1^H-NMR (500.16 MHz, DMSO-*d*_6_): δ 10.85 (br, 1H, NH), 8.45 (s, 1H, NH), 7.92 (d, *J* = 8.1 Hz, 1H, H5), 7.43 (t, *J* = 8.0 Hz, 1H, H7), 7.27 (d, *J* = 9.2 Hz, 1H, H8), 7.14 (t, *J* = 9.2 Hz, 1H, H6), 6.17 (s, 2H, NH_2_), 2.05 (br, 2H, CH_2_b), 1.44 (m, 2H, CH_2_c), 1.33-1.11 (m, 20H, CH_2_), 0.75 (br, 3H, CH_3_). ^13^C-NMR (100.52 MHz) δ 170.72 (NCO), 166.88 (C4O), 148.85 (NC2N), 145.59 (C3N), 134.81 (C10), 131.09 (C7), 125.13 (C6), 123.42 (C5), 121.92(C8), 116.48 (C9), 34.21 (CH_2_ b), 31.88 (CH_2_), 29.94 (CH_2_), 29.71 (CH_2_), 29.41 (CH_2_), 29.39 (CH_2_), 29.31 (CH_2_), 28.98 (CH_2_), 28.95 (CH_2_), 28.89 (CH_2_), 24.85 (CH_2_), 22.35 (CH_2_), 14.31 (CH_3_).MS (FAB, *m*/*z*): 386 (M^+^ + 1, 100%); Elemental analysis: C 71.61%, H 9.27%, N 10.72 % Calc. for C_23_H_35_N_3_O_2_, C 71.65%, H 9.15%, N 10.90%

*N-(2-amino-4-oxo-1,4-dihydroquinolin-2-yl)hexadecanamide* (**4i**) White solid. mp > 250 °C. IR (cm^−1^): 3334 (NH_2_), 2960, 2934, 2870 (CH_2_), 1701, 1654 (C=O). ^1^H-NMR (500.16 MHz, DMSO-*d*_6_): δ 10.94 (s, 1H, NH)8.47 (s, 1H, NH), 7.92 (s, 1H, H5), 7.43 (br, 1H, H7), 7.40 (s, 1H, H8), 7.27 (t, *J* = 9.2 Hz, 1H, H6), 6.14 (s, 2H, NH), 2.05 (s, 2H, CH_2_b), 1.54 (m, 2H, CH_2_c), 1.36-1.03 (m, 24H, CH_2_), 0.81 (br, 3H, CH_3_). ^13^C-NMR (100.52 MHz) δ 171.27 (NCO), 164.91 (C4O), 149.25 (NC2N), 144.94 (C3N), 134.61 (C10), 130.94 (C7), 125.02 (C6), 123.22 (C5), 121.78(C8), 116.56 (C9), 34.01 (CH_2_ b), 31.47 (CH_2_), 29.45 (2CH_2_), 29.44 (2CH_2_), 29.41 (CH_2_), 29.40 (CH_2_), 29.36 (CH_2_), 29.29 (CH_2_), 29.06 (CH_2_), 28.99 (CH_2_), 24.85 (CH_2_), 22.54 (CH_2_), 14.39 (CH_3_). MS (FAB, *m*/*z*): 414 (M^+^ + 1, 100%); Elemental analysis: C 72.41%, H 9.57%, N 10.28% Calc. for C_25_H_39_N_3_O_2_, C 72.60%, H 9.50%, N 10.16%

### 4.2. Evaluation of Biofilm Inhibition

Biofilm inhibition studies were carried out on *Staphylococcus aureus* (ATCC 33862) and *Pseudomonas aeruginosa* (ATCC 10145), which have been characterized as biofilm forming strains [41,42] using a previously reported method [43,44] and tannic acid as the known biofilm inhibitorv [29]. Briefly, preinoculums of the bacteria strains were grown in trypticase soy broth (TSB) overnight. Then, 5 mL of cultured broth was diluted with sterile TSB to an optical density of 0.1 at 600 nm, and 200 μL of this mixture was transferred to a well of a flat-bottomed polystyrene 96-well microplate (Costar 3370). 10 μL of a stock solution of the test compound dissolved in DMSO was added and mixed into the well to give the desired final concentration of compound per well. All compounds were tested in triplicate at concentrations of 20, 100, and 250 μM. Wells with only cultured TSB and sterile TSB and 10 μL of DMSO were included as positive and negative controls, respectively. Microplates were incubated for 24 h at 37 °C. Growth inhibition was determined by measuring the OD_600_ of compound-treated wells and control wells in a Biotek Epoch^TM^ absorbance microplate reader (BioTek Instruments, Inc., Winooski, VT). Liquid culture was discarded, and non-adherent bacteria were removed by washing 3 times with distilled water. Remnant biofilm was fixed with methanol and was stained with 200 μL of crystal violet (CV) at 0.1% in water for 10 min, excess CV was removed, and wells were washed again 3 times with distilled water. 100 μL of DMSO was added to dissolve CV and absorbance was quantified at 620 nm. Biofilm inhibition was determined by comparison of the absorbance of compound-treated wells to the absorbance of untreated wells as follows:

(2) $$\%\mathrm{biofilm}\;\mathrm{inhibition}=\frac{\left(A_{\mathrm{control}}-A_{\mathrm{treated}}\right)}{A_{\mathrm{control}}}\times 100$$

where A_control_ is the absorbance of untreated wells and A_treated_ is the absorbance of compound-treated wells. 

### 4.3. Inhibition of Pyocyanin Formation

The effect of compounds **3g** and **4g** on pyocyanin biosynthesis was evaluated using a previously reported methodology in triplicate [45]. Briefly, preinoculums of the bacteria strains were added to 50 mL of sterile TSB to an optical density at 600 nm (OD_600_) of 0.1. To 6 mL of this last suspension, 100 μL of a solution of **3i** or **4g** in dissolved DMSO was added to obtain a final concentration of 20 μM of the tested compound, which was incubated for 24 hrs. Cultured broth without **3g** or **4g** was used as the control. After incubation, 2 mL of chloroform was added, and the mixture was centrifuged (5000 g for 5 min). The bluish bottom layer was transferred to a new tube and 1.5 mL of 0.2 M HCl was added. The aqueous layer was separated and the absorbance at 520 μm was determined using 0.2 M HCl as the blank. The A_520_ of the untreated cultured broth was set to be 100% of the pyocyanin formation.

### 4.4. Molecular Docking

The structures of compounds **3a-i** and **4a-i** were constructed using ChemSketch (Advanced Chemistry Development, Inc., Toronto, ON, Canada) [31] and their geometry optimized using Spartan’10 [46] using MMFF//HF 6-31 G*. The docking studies were carried out using the crystal structures of PqsD (PDB code: 3H78 [39]) and LasR (PDB code: 2UV0 [37]) from *P. aeruginosa*. In the case of PqsD, the 3H78 structure is a complex of anthranilic acid with a C112A mutation of PqsD, then the alanine residue was changed to original cysteine using the mutation protocol included as part of Molegro Virtual Docker v.6.0.1 (Qiagen Bioinformatics, Aarhus, Denmark) [47,48], which interchanges the residues and performs an energy minimization to consider the potential changes induced by the amino acid mutation. Protein structures were retrieved from the Protein Data Bank [49]. All water molecules and cocrystallized ligands were removed from the downloaded structures. Docking studies were carried out in Molegro Virtual Docker v.6.0.1 (Qiagen Bioinformatics, Aarhus, Denmark) using the standard protocol referred by the fabricant. Active sites of each protein were selected as the searching sites and delimited in a 15 Å sphere. The assignments of charges on each protein and the analyzed ligands were based on standard templates as part of the program. *MolDock Optimizer* was selected as a searching algorithm with 50 runs and *Moldock score* as the scoring scheme. The poses with the lowest score (higher theoretical affinity) of each compound were selected for further analysis. To evaluate the efficacy of this procedure, the cocrystallized ligand in LasR was also included in the docking study of its respective receptor; the RMSD of the pose of the lowest energy was lower than 2 Å.

### 4.5. Molecular Dynamics Simulations

The docking poses of **4a**, **4g**, and **4i** with both PqsD and LasR were further analyzed using molecular dynamics (MD) to analyze the stability and conformational changes of the predicted complexes. The MD simulations were run using YASARA v.18.4.24 [50,51] using the AMBER 14 force field [52]. The initial structures were taken from the poses with the lowest docking score of each complex and were placed in a cell box that had an extension of 10 Å larger on each side of the protein and was filed with water molecules. Periodic boundary conditions were applied. The temperature was set at 298 K, water density to 0.997 g/cm^3^, and pH to a value of 7.4. Sodium (Na^+^) and chlorine (Cl^−^) ions were included to provide conditions that simulate a physiological solution (NaCl 0.9%). The cutoff for van der Waals interactions was 8 Å and the Particle Mesh Ewald algorithm was applied to evaluate long range electrostatic interactions. A multiple step of 2.5 fs was set, and data were collected per 100 ps to a final simulation time of 40 ns. The results were analyzed with a script included as part of the YASARA software and included the root mean square deviation (RMSD), root mean square fluctuations (RMSF), and ligand binding energy variations (using MM-PBSA calculations) along the simulation time.

## 5. Conclusions

In conclusion, we prepared some long chain amide derivatives of 2-amino-4-quinolone and 2,3-diamino-4-quinolone that inhibited biofilm formation in *P. aeruginosa.* The activity increased with the number of carbons present in the aliphatic chain, the compounds with 12 carbon atoms being the most active. This could be explained in terms of the potential affinity to LasR and PqsD as suggested from molecular docking and MD simulations. Compound **4g** is an attractive new starting point for the development of QSIs, however, solubility issues are a concern. Additional in vitro experiments to validate our hypothesis and the design of more soluble derivatives are currently underway in our research group. 

## Figures and Tables

**Table 1:**
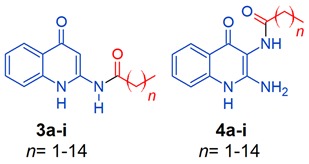
Biofilm inhibition (at 20 μM) of compounds **3a-i** and **4a-i** in *S. aureus* and *P. aeruginosa* studies.

		BI (%) (±s.d.)					BI (%) (±s.d.)		
Cmp	*n*	*S.a*	*P.a*	PEI	Cmp	*n*	*S.a*	*P.a*	PEI
**3a**	1	15.3 (±2.8)	26.4 (±4.6)	1.22	**4a**	1	< 5	28.65 (±2.2)	1.24
**3b**	2	31.2 (±3.6)	30.6 (±3.9)	1.33	**4b**	2	29.8 (±5.1)	29.43 (±3.1)	1.20
**3c**	3	<5	37.5 (±2.6)	1.54	**4c**	3	<5	33.09 (±3.2)	1.28
**3d**	4	<5	36.6 (±1.7)	1.42	**4d**	4	<5	44.95 (±1.6)	1.64
**3e**	6	22.6 (±5.7)	43.8 (±3.4)	1.53	**4e**	6	<5	48.97 (±4.6)	1.62
**3f**	8	24.8 (±1.5)	50.6 (±4.0)	1.61	**4f**	8	11.9 (±4.2)	53.38 (±2.5)	1.62
**3g**	10	18.0 (±1.2)	54.2 (±4.0)	1.46	**4g**	10	15.7 (±5.8)	61.45 (±2.2)	1.72
**3h**	12	25.7 (±1.1)	52.3 (±4.2)	1.41	**4h**	12	16.8 (±4.4)	57.63 (±3.2)	1.49
**3i**	14	34.7 (±2.0)	49.8 (±1.8)	1.25	**4i**	14	14.2 (±5.4)	56.62 (±3.1)	1.37

BI (%) = percentage of biofilm inhibition in comparison to the control, S.a = *S.aureus*, P.a. = *P.aeruginosa,* percentage efficiency index.

**Table 2:** Docking scores and *c*logP values calculated for compounds **3a-i** and **4a-i**.

		MolDock Score					MolDock Score		
Ligand	BI (%)	PqsD	LasR	logP	Ligand	BI (%)	PqsD	LasR	logP
**3a**	26.36	−99.478	−101.21	2.53	**4a**	28.65	−96.4705	−104.98	1.11
**3b**	30.61	−103.646	−109.25	3.06	**4b**	29.43	−104.784	−110.48	1.64
**3c**	37.54	−105.508	−113.46	3.59	**4c**	33.09	−110.356	−116.89	2.17
**3d**	36.56	−111.982	−114.62	4.12	**4d**	44.95	−117.802	−125.21	2.70
**3e**	43.84	−125.746	−137.16	5.18	**4e**	48.97	−129.125	−142.73	3.76
**3f**	50.57	−131.982	−152.30	6.24	**4f**	53.38	−136.142	−145.82	4.82
**3g**	54.20	−124.547	−151.66	6.77	**4g**	61.45	−139.494	−152.81	5.35
**3h**	52.27	−125.681	−146.32	7.30	**4h**	57.63	−133.275	−159.79	5.88
**3i**	49.82	−147.112	−156.07	7.83	**4i**	56.62	−150.072	−181.92	6.41

**Table 3:** Average RMSD values and ligand binding energy (LBE) ^1^ calculated during MD simulations.

	LasR			PqsD		
	Average RMSD (Å)	LBE (KJ/mol)	GE ^2^	Average RMSD (Å)	LBE (KJ/mol)	GE ^2^
**4a**	1.79	7.09	--	2.55	75.69	--
**4g**	2.01	2.01	−0.56	2.38	200.44	13.86
**4i**	1.70	−11.26	−1.41	2.37	229.30	11.81
**AHL**	1.76	3.29	--	3.26	----	--

^1^ Higher values of LBE indicate better binding, ^2^ Group Efficiency relative to LBE of **4a**.

## Supplementary Materials

The following are available online at http://www.mdpi.com/1420-3049/24/2/327/s1, Figures S1 and S2: 2D ligand interaction diagrams of **4g** with PqsD and LasR; Figure S3: Representative NMR spectra.

## References

[1] Kalia V.C. (2013). Quorum sensing inhibitors: An overview. Biotechnol. Adv..

[2] Defoirdt T., Brackman G., Coenye T. (2013). Quorum sensing inhibitors: How strong is the evidence?. Trends Microbiol..

[3] Solano C., Echeverz M., Lasa I. (2014). Biofilm dispersion and quorum sensing. Curr. Opin. Microbiol..

[4] Bjarnsholt T. (2013). The role of bacterial biofilms in chronic infections. APMIS.

[5] Dickschat J.S. (2010). Quorum sensing and bacterial biofilms. Nat. Prod. Rep..

[6] De la Fuente-Núñez C., Reffuveille F., Fernández L., Hancock R.E. (2013). Bacterial biofilm development as a multicellular adaptation: Antibiotic resistance and new therapeutic strategies. Curr. Opin. Microbiol..

[7] Majik M.S., Parvatkar P.T. (2014). Next generation biofilm inhibitors for Pseudomonas aeruginosa: Synthesis and rational design approaches. Curr. Top. Med. Chem..

[8] Wilson M. (2001). Bacterial biofilms and human disease. Sci. Prog..

[9] Sharma G., Rao S., Bansal A., Dang S., Gupta S., Gabrani R. (2014). Pseudomonas aeruginosa biofilm: Potential therapeutic targets. Biologicals.

[10] Brackman G., Coenye T. (2015). Quorum sensing inhibitors as anti-biofilm agents. Curr. Pharm. Des..

[11] Brackman G., Cos P., Maes L., Nelis H.J., Coenye T. (2011). Quorum sensing inhibitors increase the susceptibility of bacterial biofilms to antibiotics in vitro and in vivo. Antimicrob. Agents Chemother..

[12] Zhu J., Kaufmann G.F. (2013). Quo vadis quorum quenching?. Curr. Opin. Pharmacol..

[13] Blackwell H.E., Fuqua C. (2011). Introduction to Bacterial Signals and Chemical Communication. Chem. Rev..

[14] Lee J., Zhang L. (2015). The hierarchy quorum sensing network in Pseudomonas aeruginosa. Protein Cell.

[15] Storz M.P., Maurer C.K., Zimmer C., Wagner N., Brengel C., de Jong J.C., Lucas S., Müsken M., Häussler S., Steinbach A. (2012). Validation of PqsD as an Anti-biofilm Target in Pseudomonas aeruginosa by Development of Small-Molecule Inhibitors. J. Am. Chem. Soc..

[16] O’Brien K.T., Noto J.G., Nichols-O’Neill L., Perez L.J. (2015). Potent Irreversible Inhibitors of LasR Quorum Sensing in *Pseudomonas aeruginosa*. ACS Med. Chem. Lett..

[17] Kalia M., Singh P.K., Yadav V.K., Yadav B.S., Sharma D., Narvi S.S., Mani A., Agarwal V. (2017). Structure based virtual screening for identification of potential quorum sensing inhibitors against LasR master regulator in Pseudomonas aeruginosa. Microb. Pathog..

[18] Sahner J.H., Empting M., Kamal A., Weidel E., Groh M., Börger C., Hartmann R.W. (2015). Exploring the chemical space of ureidothiophene-2-carboxylic acids as inhibitors of the quorum sensing enzyme PqsD from Pseudomonas aeruginosa. Eur. J. Med. Chem..

[19] Sahner J.H., Brengel C., Storz M.P., Groh M., Plaza A., Müller R., Hartmann R.W. (2013). Combining in Silico and Biophysical Methods for the Development of Pseudomonas aeruginosa Quorum Sensing Inhibitors: An Alternative Approach for Structure-Based Drug Design. J. Med. Chem..

[20] Zhou Z., Ma S. (2017). Recent Advances in the Discovery of PqsD Inhibitors as Antimicrobial Agents. ChemMedChem.

[21] Schütz C., Empting M. (2018). Targeting the *Pseudomonas* quinolone signal quorum sensing system for the discovery of novel anti-infective pathoblockers. Beilstein J. Org. Chem..

[22] Basak A., Abouelhassan Y., Kim Y.S., Norwood V.M., Jin S., Huigens R.W. (2018). Halogenated quinolines bearing polar functionality at the 2-position: Identification of new antibacterial agents with enhanced activity against Staphylococcus epidermidis. Eur. J. Med. Chem..

[23] Zaheer Z., Khan F.A.K., Sangshetti J.N., Patil R.H., Lohar K.S. (2016). Novel amalgamation of phthalazine–quinolines as biofilm inhibitors: One-pot synthesis, biological evaluation and in silico ADME prediction with favorable metabolic fate. Bioorg. Med. Chem. Lett..

[24] Zuo R., Garrison A.T., Basak A., Zhang P., Huigens R.W., Ding Y. (2016). In vitro antifungal and antibiofilm activities of halogenated quinoline analogues against Candida albicans and Cryptococcus neoformans. Int. J. Antimicrob. Agents.

[25] Abouelhassan Y., Garrison A.T., Burch G.M., Wong W., Norwood V.M., Huigens R.W. (2014). Discovery of quinoline small molecules with potent dispersal activity against methicillin-resistant Staphylococcus aureus and Staphylococcus epidermidis biofilms using a scaffold hopping strategy. Bioorg. Med. Chem. Lett..

[26] Speck-Planche A., Kleandrova V.V., Scotti M.T. (2012). Fragment-based approach for the in silico discovery of multi-target insecticides. Chemom. Intell. Lab. Syst..

[27] Aleksić I., Šegan S., Andrić F., Zlatović M., Moric I., Opsenica D.M., Senerovic L. (2017). Long-Chain 4-Aminoquinolines as Quorum Sensing Inhibitors in *Serratia marcescens* and *Pseudomonas aeruginosa*. ACS Chem. Biol..

[28] Maurino-Reyes E.D.J., Gonzalez-Rodriguez E., Reyes-Rangel F., Lira-Rocha A., Loza-Mejia M.A. (2016). A direct synthetic route to fused tricyclic quinolones from 2,3-diaminoquinolin-4(1H)one. Heterocycl. Commun..

[29] Peeters E., Hooyberghs G., Robijns S., Waldrant K., De Weerdt A., Delattin N., Liebens V., Kucharíková S., Tournu H., Verstraeten N. (2016). Modulation of the Substitution Pattern of 5-Aryl-2-Aminoimidazoles Allows Fine-Tuning of Their Antibiofilm Activity Spectrum and Toxicity. Antimicrob. Agents Chemother..

[30] Abad-Zapatero C., Metz J.T. (2005). Ligand efficiency indices as guideposts for drug discovery. Drug Discov. Today.

[31] Advanced Chemistry Development ChemSketch. www.acdlabs.com.

[32] Verdonk M.L., Rees D.C. (2008). Group Efficiency: A Guideline for Hits-to-Leads Chemistry. ChemMedChem.

[33] Cavalluzzi M.M., Mangiatordi G.F., Nicolotti O., Lentini G. (2017). Ligand efficiency metrics in drug discovery: The pros and cons from a practical perspective. Expert Opin. Drug Discov..

[34] Park S., Kim H.-S., Ok K., Kim Y., Park H.-D., Byun Y. (2015). Design, synthesis and biological evaluation of 4-(alkyloxy)-6-methyl-2H-pyran-2-one derivatives as quorum sensing inhibitors. Bioorg. Med. Chem. Lett..

[35] Hodgkinson J.T., Galloway W.R.J.D., Wright M., Mati I.K., Nicholson R.L., Welch M., Spring D.R. (2012). Design, synthesis and biological evaluation of non-natural modulators of quorum sensing in Pseudomonas aeruginosa. Org. Biomol. Chem..

[36] Leeson P.D., Springthorpe B. (2007). The influence of drug-like concepts on decision-making in medicinal chemistry. Nat. Rev. Drug Discov..

[37] Bottomley M.J., Muraglia E., Bazzo R., Carfì A. (2007). Molecular insights into quorum sensing in the human pathogen Pseudomonas aeruginosa from the structure of the virulence regulator LasR bound to its autoinducer. J. Biol. Chem..

[38] O’Reilly M.C., Dong S.-H., Rossi F.M., Karlen K.M., Kumar R.S., Nair S.K., Blackwell H.E. (2018). Structural and Biochemical Studies of Non-native Agonists of the LasR Quorum-Sensing Receptor Reveal an L3 Loop “Out” Conformation for LasR. Cell Chem. Biol..

[39] Bera A.K., Atanasova V., Robinson H., Eisenstein E., Coleman J.P., Pesci E.C., Parsons J.F. (2009). Structure of PqsD, a *Pseudomonas* Quinolone Signal Biosynthetic Enzyme, in Complex with Anthranilate. Biochemistry.

[40] Steinbach A., Maurer C.K., Weidel E., Henn C., Brengel C., Hartmann R.W., Negri M. (2013). Molecular basis of HHQ biosynthesis: Molecular dynamics simulations, enzyme kinetic and surface plasmon resonance studies. BMC Biophys..

[41] Cruciata M., Gaglio R., Scatassa M.L., Sala G., Cardamone C., Palmeri M., Moschetti G., La Mantia T., Settanni L. (2018). Formation and Characterization of Early Bacterial Biofilms on Different Wood Typologies Applied in Dairy Production. Appl. Environ. Microbiol..

[42] Pires D., Sillankorva S., Faustino A., Azeredo J. (2011). Use of newly isolated phages for control of Pseudomonas aeruginosa PAO1 and ATCC 10145 biofilms. Res. Microbiol..

[43] Kwasny S.M., Opperman T.J. (2010). Static biofilm cultures of Gram-positive pathogens grown in a microtiter format used for anti-biofilm drug discovery. Curr. Protoc. Pharmacol..

[44] Martínez Díaz Y., Vanegas Laverde G., Reina Gamba L., Mayorga Wandurraga H., Arévalo-Ferro C., Ramos Rodríguez F., Duque Beltrán C., Castellanos Hernández L. (2015). Biofilm inhibition activity of compounds isolated from two Eunicea species collected at the Caribbean Sea. Rev. Bras. Farmacogn..

[45] Tapia-Rodriguez M.R., Hernandez-Mendoza A., Gonzalez-Aguilar G.A., Martinez-Tellez M.A., Martins C.M., Ayala-Zavala J.F. (2017). Carvacrol as potential quorum sensing inhibitor of Pseudomonas aeruginosa and biofilm production on stainless steel surfaces. Food Control..

[46] Wavefunction Inc Spartan ’10. www.wavefun.com.

[47] Thomsen R., Christensen M.H. (2006). MolDock:  A New Technique for High-Accuracy Molecular Docking. J. Med. Chem..

[48] CLC Bio Molegro Virtual Docker. https://www.qiagenbioinformatics.com/.

[49] Berman H.M., Westbrook J., Feng Z., Gilliland G., Bhat T.N., Weissig H., Shindyalov I.N., Bourne P.E. (2000). The Protein Data Bank. Nucleic Acids Res..

[50] Krieger E., Vriend G. (2015). New ways to boost molecular dynamics simulations. J. Comput. Chem..

[51] Yasara Dynamics. www.yasara.org.

[52] Maier J.A., Martinez C., Kasavajhala K., Wickstrom L., Hauser K.E., Simmerling C. (2015). ff14SB: Improving the Accuracy of Protein Side Chain and Backbone Parameters from ff99SB. J. Chem. Theory Comput..

