# Supplementary material for: Synthesis, In Silico, and In Vitro Evaluation of Long Chain Alkyl Amides from 2-Amino-4-Quinolone Derivatives as Biofilm Inhibitors
Source: Molecules. 2019 Jan 17;24(2):327. doi: 10.3390/molecules24020327 (PMC6359591; doi:10.3390/molecules24020327)
Supplement: Supplementary file 1 [file molecules-24-00327-s001.pdf]

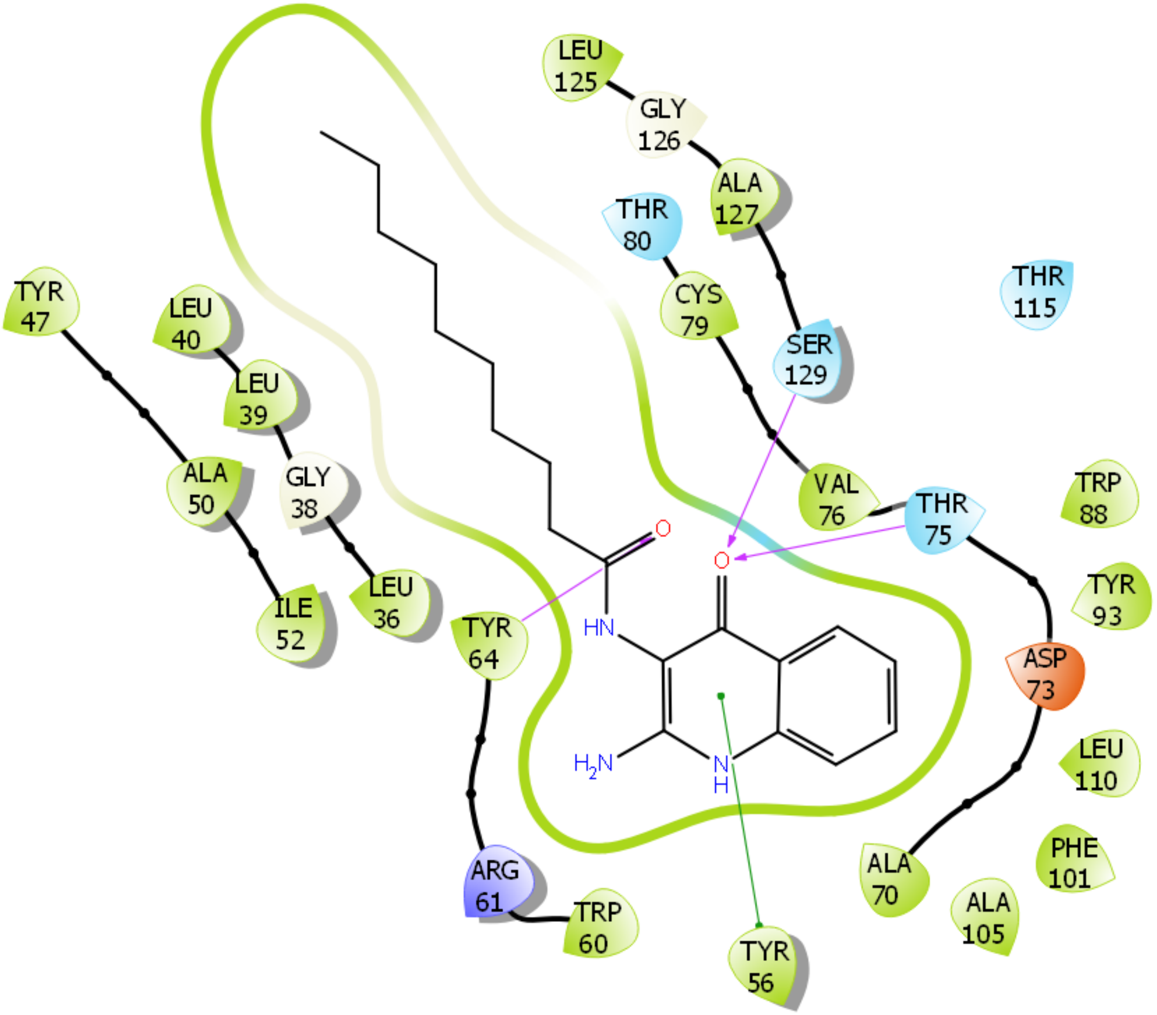

Figure S1. 2D ligand interaction diagram for compound **4g** with LasR active site.

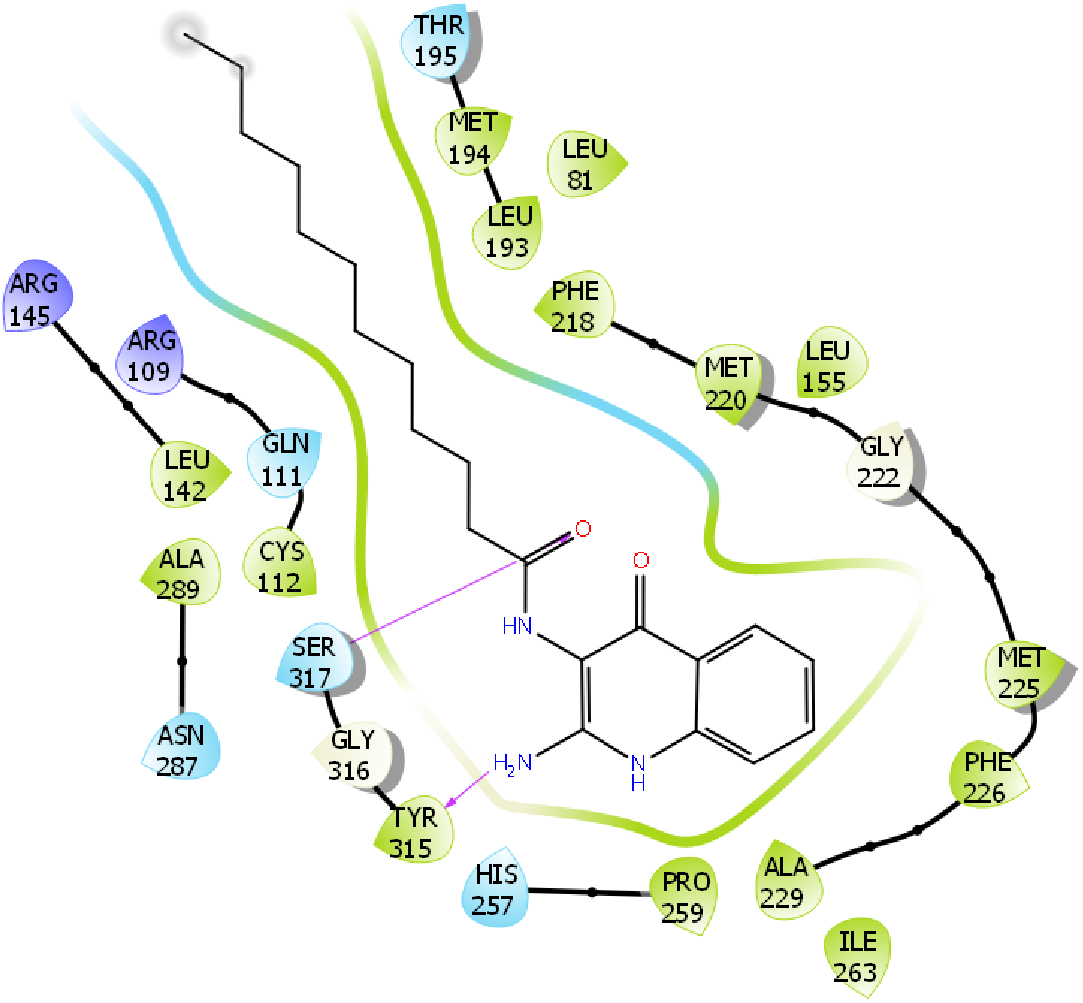

Figure S2. 2D ligand interaction diagram for compound **4g** with LasR active site.

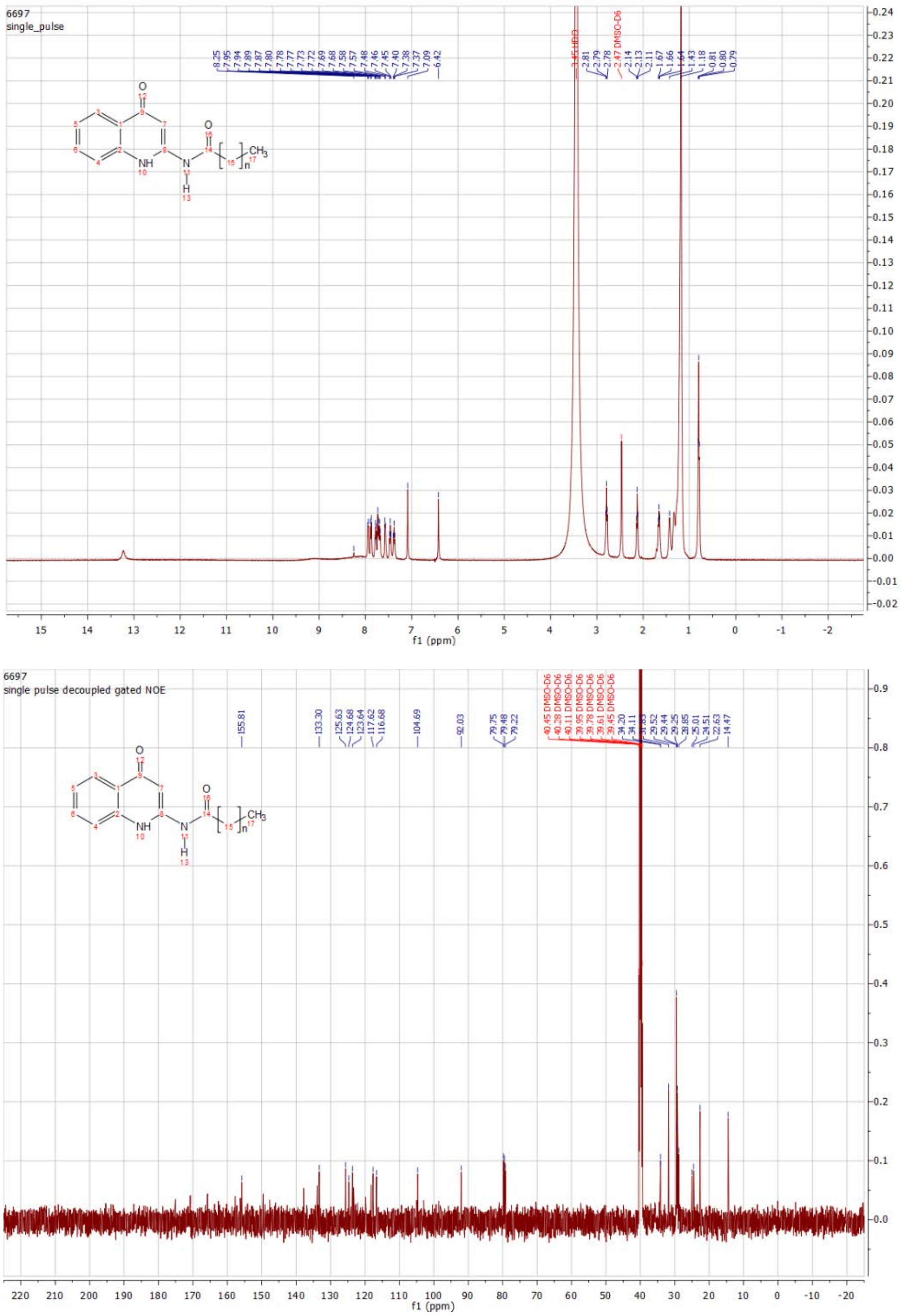

Figure S3. Typical NMR spectra.

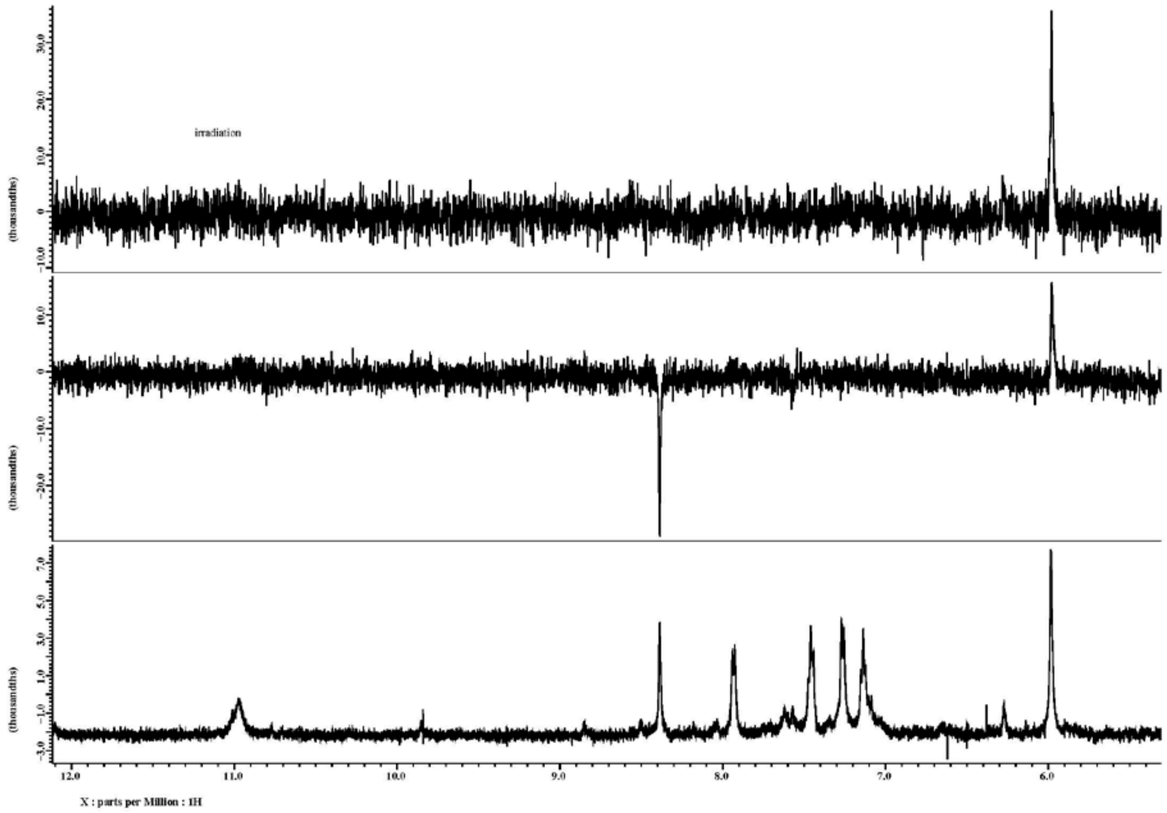

Figure S4. 1D NOE spectra generated during selective irradiation of protons of 2-amino group.
